# Study Protocol: Adolescents of Ukraine During the Russian Invasion (AUDRI) Cohort

**DOI:** 10.1186/s12889-023-16070-3

**Published:** 2023-07-12

**Authors:** Ryunosuke Goto, Irina Pinchuk, Oleksiy Kolodezhny, Nataliia Pimenova, Norbert Skokauskas

**Affiliations:** 1 Department of Pediatrics, The University of Tokyo Hospital, Tokyo, Japan; 2 Institute of Psychiatry, Taras Shevchenko National University of Kyiv, Kyiv, Ukraine; 3 Regional Centre for Child and Youth Mental Health and Child Welfare, Norwegian University of Science and Technology, Trondheim, Norway; 4 Chair, Child and Adolescent Psychiatry Section, World Psychiatric Association, Geneva, Switzerland

**Keywords:** Humanitarian health, Global health, Prospective cohort, Epidemiology, Child and adolescent psychiatry, Child and adolescent mental health, Psychiatry

## Abstract

**Background:**

Since February 14, 2022, Ukraine has once again been under attack by the Russian forces, putting the nation in one of the biggest emergencies in Europe since World War II. This puts Ukrainians at high risk of psychiatric disorders, amidst unseen attacks on infrastructure that have put massive strain on Ukraine’s mental health services. Despite this, the prevalence of psychiatric disorders among adolescents and their changes over time have not yet been documented in Ukraine during the invasion. More generally, there is a need to more comprehensively uncover the long-term consequences of war on youth, especially their risks and protective factors.

**Methods:**

The Adolescents of Ukraine During the Russian Invasion (AUDRI) Cohort is the largest cohort of war-affected Ukrainian adolescents. We will recruit adolescents aged 15 to 18 years attending any school in Ukraine. Data collection will start early 2023, and will be held via online questionnaires every six months during the war as well as after the war has terminated. We will use several well-validated tools to screen for PTSD, depression, anxiety, substance use disorder, and eating disorders. In addition, we will ask participants about possible risks and protective factors of their mental health including resilience and social capital. Using the cohort, we will evaluate the trends in psychiatric disorder prevalence among adolescents in Ukraine over time and evaluate risks and protective factors of adolescents’ mental health.

**Discussion:**

The AUDRI Cohort will provide a unique opportunity to learn more about trauma and resilience among youth in conflict settings, in addition to aiding international efforts to save the mental health of youth in Ukraine. At-risk adolescents identified from our study can directly become beneficiaries of targeted intervention themselves. Building evidence on the mental health of adolescents is especially valuable, as protecting the mental health of war-affected adolescents could help rebuild society and have positive consequences for generations to come.

## Background

Starting in 24 February 2022, Ukraine, one the largest countries in Europe, has been facing one of the biggest emergencies in Europe since World War II [1]. Of Ukraine’s 43.7 million people, [2] the United Nations High Commissioner for Refugees (UNHCR) reports that 7.9 million have sought refuge in neighboring countries, [3] while the International Organization for Migration reports that an additional 6.5 million are internally displaced [4].

According to the United Nations International Children’s Emergency Fund (UNICEF), as of June 1, 2022, three million children inside Ukraine and over 2.2 million children in refugee-hosting countries were in need of humanitarian assistance, [5] and almost two out of every three children have been displaced by fighting. Arguably, negative health sequelae of war on the civilian population could affect society for years to come, especially the mental health of children: there have been reports of delayed socioemotional development in children as young as three to four years old [6] and a high prevalence of mental disorders such as depression, anxiety, bipolar disorder, and PTSD among war-affected populations, [7] secondary to exposure to violence and displacement and loss of usual routines at home and at school [6, 8, 9]. In Ukraine under Russian invasion, the increased need for mental health care is amidst the unseen attacks on infrastructure (and the resulting energy cuts and water disruptions) that have put massive strain on mental health services in Ukraine; this includes shortages of staff and medications, burnout among staff, and destructions of psychiatric hospitals across the nation [10, 11]. However, despite calls for action to address the mental health burden of the war in Ukraine, the prevalence of psychiatric disorders and their changes over time have not yet been documented [9]. There is a need for a more comprehensive understanding of the mental health consequences of the war-affected civilian population, their changes over time, and the risks and protective factors, especially in the setting of the invasion of Ukraine.

The consequences of war have been previously studied, especially among veterans, with former child soldiers and veterans experiencing large burdens of mental disorders such as post-traumatic stress disorder (PTSD) and depression [12–15]. However, the long-term health consequences of war on the civilian population, especially children, and their risks and protective factors have yet to be fully uncovered. Importantly, the consequences of war on mental health of civilians have not been well-documented in armed conflicts as large as the ongoing conflict in Ukraine. The reasons for the dearth of longitudinal data on the civilian population during and after exposure to war could be multifactorial, but a large portion could lie in the difficulty in data collection. With damaged infrastructure, directly accessing the war-affected population is extremely challenging. We will use digital technologies to overcome this challenge. Even in low- and middle-income countries or war-affected areas, smartphone possession and access to the internet has become increasingly high [16]. As of 2019, 66% of Ukrainians have smartphones (a large increase compared to 45% in 2018) and 93% have access to the internet, and is now presumably in the hands of many more Ukrainians today given the current pace of penetration led by the efforts of the Ministry of Digital Transformation [17, 18]. Furthermore, though the current Russian invasion of Ukraine has damaged the infrastructure of the nation, internet access has largely remained largely intact with international efforts to maintain access [19]. Thus, using technology to our advantage, we aim to build a large-scale and sustainable cohort: we will utilize solely web-based surveys to allow us to continually monitor the mental health of war-exposed participants over time, even if they move to another area or become refugees.

The AUDRI Cohort will give us a unique opportunity to learn more about trauma and resilience among youth in conflict settings. Collecting data on adolescents is especially valuable, as protecting the mental health of war-affected adolescents could have positive consequences for generations to come and help rebuild society [9]. Our cohort could help identify important risk and protective factors, and at-risk adolescents can also directly become targets of intervention themselves; this is helpful for policymakers, as it is extremely difficult to intervene on all youth in Ukraine given the cost and accessibility, and targeted interventions are often the most feasible and effective option. Building evidence-based targeted interventions requires large samples (from which effects of subgroups are deduced), and our cohort could serve as the basis for building such evidence. Our findings could lead to not only international aid for Ukraine during the crisis, but also serve as a valuable evidence base for future humanitarian crises that require mental health support for youth.

## Methods/Design

### Study design

The Adolescents of Ukraine During the Russian Invasion (AUDRI) Cohort is a large cohort of war-affected Ukrainian adolescents.

### Participant recruitment

Participant recruitment will take place in all regions of Ukraine, where youth aged 15 to 18 years attending any school in Ukraine will be recruited. The maximum expected number of participants is about 400,000 youth, with the aim of obtaining a representative sample of all adolescents attending school in Ukraine. Data collection will start in early 2023, and will be held every six months during the war as well as after the war has terminated.

We will contact schools in all regions of Ukraine, who will distribute the links and/or QR codes of the questionnaire to eligible students through their electronic mailing systems. In the follow-up phases, participants will be sent links to access the questionnaire at appropriate timings. In each phase, both participants and their legal parent/guardian will be asked to read a description of the study and ethical considerations and complete an informed consent form. All questionnaires will be self-administered, conducted in Ukrainian, and answered online.

We will use Qualtrics to build and distribute the questionnaire, store the data, link individuals’ data across waves, and extract the de-identified data for analyses. Qualtrics ensures that the privacy of participants is protected, complying with the General Data Protection Regulation and the California Consumer Privacy Act [20]. Using Qualtrics, we will be able to send invitations to follow-up studies to participants and link individual data across surveys. During this process, the researchers will not access the participants’ personal contact information; recruitment and data linkage will be done anonymously on Qualtrics.

### Measures

A full list of the measures used in the AUDRI cohort and the waves that they will be administered is shown in the Table 1. In the baseline data collection, we will collect demographic data, including age, self-identified gender, sex, and educational level of the participants’ parents. Participants will also be asked whether they have had any learning difficulties as a child or have attended special education classes, in addition to whether they have been diagnosed with autism spectrum disorder (ASD) and/or attention deficit hyperactivity disorder (ADHD). In each wave, the participants will be asked about their displacement status and current (and, if displaced, previous) place of residence, as well as whether they have been separated from their parents due to the war. In addition, we will use the Social Capital Questionnaire for Adolescent Students (SCQ-AS) [21] to assess social capital and the Connor-Davidson Resilience Scale (CD-RISC) [22] to assess resilience, as factors of potential importance in association with psychiatric disease prevention.

**Table 1:** AUDRI Cohort data collection and measures

Measures	Baseline (Early 2023)	Follow-up (Every 6 months)
Demographic information (age, self-identified gender, sex, education level of parents)	x	
Place of residence	x	x
Displacement status	x	x
Separation from parents due to the war	x	x
Learning difficulties and developmental disorders (receipt of special education, diagnosis of ASD/ADHD)	x	
Social Capital Questionnaire for Adolescent Students (SCQ-AS) [21]	x	x
Connor-Davidson Resilience Scale (CD-RISC) [22]	x	x
Child and Adolescent Trauma Screen (CATS) [23]	x	x
Patient Health Questionnaire-9 for Adolescents (PHQ-A) [25]	x	x
Generalized Anxiety Disorder 7 (GAD-7) [26]	x	x
CRAFFT (for substance-related risks and problems) [31]	x	x
SCOFF (for eating disorders) [32]	x	x

In each wave, we will use several well-validated tools to screen for PTSD, depression, anxiety, substance use disorder, and eating disorders. The Child and Adolescent Trauma Screen (CATS), a well-validated tool for evaluating post-traumatic stress symptoms among youth, will be used to screen for PTSD, using recommended cutoff scores [23, 24]. The CATS contains questions on being around war or witnessing violence or death, from which we will extract whether the participant has been exposed to war. We will use Patient Health Questionnaire-9 for Adolescents (PHQ-A) [25] to screen for depression. To screen for anxiety, we will use the Generalized Anxiety Disorder 7 (GAD-7), [26] which has been validated for use on adolescents in several studies [27, 28]. The self-administered CRAFFT 2.1 will be used to screen for substance use disorders [29–31]. Finally, we will use the SCOFF, [32] which has been validated for use on adolescents, [33] to screen for eating disorders.

### Statistical analyses

We will use several methods to examine the prevalence of psychiatric disorders over time and their relationship with exposure to war, as well as their risk and protective factors. First, we will plot the changes in the prevalence of psychiatric disorders over time. Next, we will use causal inference methods (e.g., using marginal structural models) [34] to examine the associations with psychiatric disorders and exposure to war, resilience, and social capital, among other variables. We chose these as some variables may be time-varying (e.g., resilience and social capital). This will allow us to identify risk and protective factors of psychiatric disease during war as well as their effect sizes. Finally, we will construct models, including regression models and machine learning models, to explore the risk and protective factors of psychiatric disease during war in greater detail and to predict the onset of psychiatric disease based on individual-level characteristics.

### Ethical and regulatory considerations

The study will be conducted in accordance with the principles of the Declaration of Helsinki. The Ethics Committees of the Institute of Psychiatry at the Taras Shevchenko National University of Kyiv (No. 7/23/11/2022) and the University of Tokyo (No. 2023022NI) approved this study. The study staff will ensure the participants’ anonymity is protected, and the email addresses used to merge data across time points will be deleted immediately after linkage has been completed. All documents will be stored online securely and will only be accessible by authorized staff.

### Publication policy

The results of this study will be published in academic journals and conferences. A policy paper will be prepared for the Office of the President of Ukraine for further dissemination to domestic and international stakeholders (e.g., international organizations and governments) as well as the public. The study will also be widely disseminated in social media platforms (e.g., Twitter) so that the study participants, their family, and domestic and international stakeholders could easily access the findings of this study.

## Discussion

The AUDRI cohort will evaluate the mental health of adolescents using a longitudinal design. A major strength of this study is that it not only provides information on the prevalence of major psychiatric disorders in war-affected Ukraine, but also identify important risks and protective factors during a humanitarian crisis. Such data are extremely difficult to accumulate in a humanitarian setting, given the difficulty in accessing affected sites. Our surveys will be solely internet-based, and adolescents will be able to participate wherever they are. We believe this is essential, as war-affected populations are often displaced multiple times during war, making physical access to the same individuals (which is necessary in a cohort, when data are not collected online) quite challenging. Ultimately, using technology to our advantage, we plan to build a large, sustainable cohort to help protect the future of Ukraine and its people.

## Data Availability

To protect the privacy of the participants, the datasets generated and/or analysed during the current study will not be publicly available. Anyone seeking to access the data should contact the corresponding author.

## Abbreviations

UNHCR: United Nations High Commissioner for Refugees
UNICEF: United Nations International Children’s Emergency Fund
PTSD: Post-traumatic stress disorder
AUDRI: Adolescents of Ukraine During the Russian Invasion
ASD: Autism spectrum disorder
ADHD: Attention deficit hyperactivity disorder
SCQ-AS: Social Capital Questionnaire for Adolescent Students
CD-RISC: Connor-Davidson Resilience Scale
CATS: Child and Adolescent Trauma Screen
PHQ-A: Patient Health Questionnaire-9 for Adolescents
GAD-7: Generalized Anxiety Disorder 7
